# Is Diastasis Recti Abdominis Rehabilitation after Childbirth Able to Prevent the Onset of Stress Urinary Incontinence? A Case-Control Study

**DOI:** 10.3390/medicina59122182

**Published:** 2023-12-15

**Authors:** Andrea Braga, Livia Galli, Giorgio Caccia, Giulia Amato, Andrea Papadia, Marco Torella, Stefano Salvatore, Chiara Scancarello, Yoav Baruch, Maurizio Serati

**Affiliations:** 1 Department of Obstetrics and Gynecology, EOC-Beata Vergine Hospital, 6850 Mendrisio, Switzerland; giorgio.caccia@eoc.ch (G.C.); giulia.amato@eoc.ch (G.A.); 2 Faculty of Biomedical Sciences, Università della Svizzera Italiana, 6900 Lugano, Switzerland; galli.livia@gmail.com (L.G.); andrea.papadia@eoc.ch (A.P.); 3 Department of Obstetrics and Gynecology, EOC-Civico Hospital, 6900 Lugano, Switzerland; 4 Department of Gyanecology, Obstetric and Reproductive Science, Second University of Naples, 80100 Naples, Italy; marcotorella@iol.it; 5 Department of Obstetrics and Gynecology, IRCSS San Raffaele Scientific Institute, 20132 Milan, Italy; stefanosalvatore@hotmail.com; 6 Department of Obstetrics and Gynecology, Del Ponte Hospital, University of Insubria, 21100 Varese, Italy; chiarascanca@gmail.com (C.S.); mauserati@hotmail.com (M.S.); 7 Department of Obstetrics and Gynecology, Tel Aviv Medical Center, Tel Aviv University, Tel Aviv 6997801, Israel; yoavi100@gmail.com

**Keywords:** pelvic floor dysfunctions, abdominal wall, urinary incontinence, stress urinary incontinence, diastasis recti abdominis

## Abstract

*Background and Objectives:* Pelvic floor muscle training (PFMT) represent the first-line approach to pelvic floor dysfunctions (PFDs). Recently, studies have shown a synergy between the pelvic floor and abdominal muscles, hypothesizing that the anatomical and functional integrity of the abdominal wall plays a role in the prevention of pelvic floor disorders. Some studies have shown a significant correlation between diastasis recti abdominis (DRA) and stress urinary incontinence (SUI). Nevertheless, the evidence reported in the literature is controversial and based on low-quality data. The aim of the study is to clarify whether DRA-specific abdominal rehabilitation is needed in women with SUI after childbirth. *Materials and Methods:* All consecutive women who had at least one delivery and complained of symptoms of pure SUI that were urodynamically proven were considered for the study. The group of symptomatic patients was compared with a series of consecutive women, identified during the same study period, without any symptoms of SUI. In both groups, we measured the inter-rectal distance (IRD) with an ultrasound scanner above and below the navel. *Results:* A total of 102 women eligible for the study group and 100 women who did not report any symptoms of SUI were enrolled. The inter-rectal distance above the umbilicus showed no significant difference between the two groups (2.12 ± 0.98 vs. 2.1 ± 0.77; *p* = 0.94). In contrast, the data from the sub-umbilical measurements showed a significant difference. Surprisingly, the asymptomatic group showed significantly greater (0.98 ± 0.9 vs. 1.33 ± 0.87 *p*-value: 0.009) IRD compared to the symptomatic group. *Conclusions:* The study shows that DRA is not a risk factor for SUI in women after childbirth. Therefore, specific abdominal wall rehabilitation after childbirth does not seem to be indicated.

## 1. Introduction

Pelvic floor dysfunctions are very common in women and affect their quality of life significantly. Studies have shown a high prevalence between 23 and 46% [1,2] and obstetric factors are recognized as the most important risk factors for their development [3]. In a recent prospective study, which aimed to investigate the prevalence of PFDs after delivery, Soligo et al. reported a prevalence of 34.7% at 3 months postpartum [4]. Another prospective study observed a prevalence of de novo urinary incontinence (UI), anal incontinence and sexual dysfunctions of 27%, 7.1% and 23.8%, respectively, at 6 months postpartum [5]. Stress urinary incontinence (SUI), defined by the International Urogynecological Association (IUGA)/International Continence Society (ICS) joint report [6], as an involuntary loss of urine following physical exertion or sneezing or coughing, represents the most common type of UI, especially after childbirth. Thom and Rortveit [7] in a systematic review on the prevalence of urinary incontinence in the first year postpartum, showed that SUI was almost four times more common than urge incontinence in primiparous women, while for all parous women, urge incontinence was substantially more common, but still lower than SUI. Furthermore, they found a double prevalence in the vaginal delivery group (31%, 95% CI 30–33%) compared to the cesarean section group (15%, 95% CI 11–18%), confirming that vaginal birth may be considered the primum movens leading to an increased risk of developing PFDs. In fact, vaginal delivery is a phenomenon involving various structures such as muscles, nerves and connective tissue, during the descent of the fetal head. The activation and stretching of these structures can influence the outcomes of vaginal delivery and the occurrence of PFDs. In particular, the duration of the second stage of labor, fetal weight and the use of vacuum extractor are considered the main predictors of the onset of UI. 

Several studies in the literature, suggest that women with UI may benefit from pelvic floor muscle training (PFMT) and other rehabilitative care, during the pre and postpartum period. As recommended by the most important urological and urogynaecological societies, pelvic floor muscle training represent the first-line approach to PFDs [8]. In addition, the 7th recommendation of International Consultation on Incontinence (ICI) 2023 recommends to all continent pregnant women to offer a supervised PFMT including routine health professional contact (Grade of Recommendation: A) [9]. Recently, studies have shown a synergy between the pelvic floor and abdominal muscles [10], hypothesizing that the anatomical and functional integrity of the abdominal wall plays a role in the prevention of PFDs [11,12,13]. Smith et al. [14] evaluated using electromyographic activity whether activity of the pelvic floor and abdominal muscles differs between continent and incontinent women in response to a postural perturbation with a moderately full or empty bladder. The authors found that women with UI had increased pelvic floor and abdominal muscle activity associated with postural perturbations. These results are contrary to the hypothesis that UI is related to poor pelvic floor muscle activity and suggest that abdominal wall rehabilitation may be helpful in treating this problem. 

According to some authors, diastasis recti abdominis, defined as the enlargement of the distance between the edges of the recti abdominis at the level of the linea alba [15], could have an impact on the development of PFDs. Nevertheless, the evidence reported in the literature is controversial and based on low-quality data [16]. A systematic review by Fuentes et al. on the topic of self-reported symptoms in women with DRA, showed a significant correlation between DRA and quality of life, physical function, pain and body image, but only a heterogeneous correlation with PFDs [17]. Spitznagle et al. showed in a retrospective review that women with DRA had a 1.79 times greater risk of having a PFDs compared to women without DRA. Remarkably, the data correlated with an increasing number of PFDs and DRA compared with the group without DRA [18]. In addition, two case studies noted resolution of preexisting UI after abdominoplasty [19,20]. In contrast, Wang et al. found no significant association between women with PFD and DRA in their cross-sectional study [12]. Furthermore, in a retrospective cohort study examining 229 women, Fei et al. showed no significant association between PFDs and DRA [21]. Overall, there is little clarity on the contribution of DRA to the explanation of postpartum pelvic floor disorders, and there is little guidance in the literature for health care providers on the management of this problem.

For these reasons, the aim of the study is to clarify whether DRA-specific abdominal rehabilitation is needed in women with UI after childbirth.

## 2. Materials and Methods

**Figure 1:**
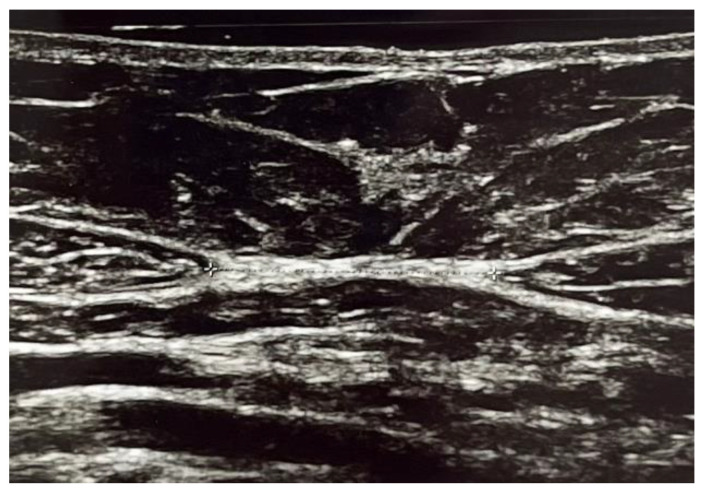
Ultrasound image of supraumbilical inter-rectal space.

**Figure 2:**
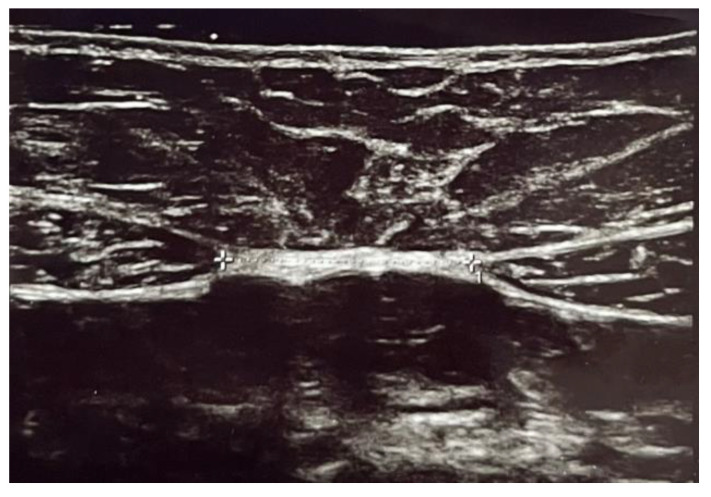
Ultrasound image of subumbilical inter-rectal space.

This case–control study was carried out at the Urogynecological Unit of the Department of Obstetrics and Gynecology, Mendrisio (CH), between August 2021 and February 2023. All consecutive women who complained symptoms of pure SUI according to the International Urogynecological Association/International Continence Society terminology for Female Floor Dysfunctions [6], confirmed urodynamically and who had at least one delivery, were considered for the study and allocated in group 1. Exclusion criteria were nulliparous women, previous abdominal surgery, Body Mass Index (BMI) > 30 kg/m^2^, postvoid residual (PVR) > 100 mL, previous weight loss > 10 kg, presence of abdominal hernia, pathological connective tissue laxity, previous history of radical pelvic surgery, psychiatric and neurologic disorders. At the first visit, patients underwent medical history collection, physical examination, urine analysis and ultrasound evaluation. Physical examination was performed with the patient in the lithotomic position and POP was described during maximal Valsalva according to the pelvic-organ prolapse quantification (POP-Q) system [21]. The SUI test was performed in the upright position with a full bladder (ultrasonographic measurement > 300 mL). The patient had to strongly cough one, three and five times in this position. If urine was lost, the test was considered positive. All patients also completed the following validate questionnaires: Urogenital Distress Inventory Short Form (UDI-6), Incontinence Impact Questionnaire Short Form (IIQ-7) and International Consultation on Incontinence Questionnaire Short Form (ICIQ-SF) [22]. Additionally, to gauge the severity of SUI symptom, Visual Analogue Scale (VAS) scores were obtained in every woman. This involved using a self-administered, Likert-type scale ranging from 0 to 10, where the patients rate their discomfort of different symptoms. A score of 0 indicates no discomfort, while a score of 10 represents the highest degree of discomfort possible. All women were evaluated with urodynamic studies (including uroflowmetry, filling cystometry, Valsalva leak-point pressure measurement and pressure/flow study) by a trained urogynaecologist, using a standardized protocol in accordance with the best urodynamic practice guidelines of the International Continence Society [23]. The group of symptomatic patients was compared with a series of consecutive women, identified during the same study period, based on the same exclusion criteria but without any symptoms of SUI and allocated in group 2. In both groups, we evaluated the inter-rectal distance with an ultrasound scanner having 12 MHz linear probe (General Electric Healthcare Voluson E10®, GE Healthcare, Chicago, IL, USA) with the patient supine, legs fully extended and in a relaxed position without abdominal contraction and having normal breathing. The measurement was conducted in cm. All measurements of the DRA were obtained in brightness mode (B mode) by two physicians with experience in abdominal ultrasound imaging (Figure 1 and Figure 2). 

**Figure 3:**
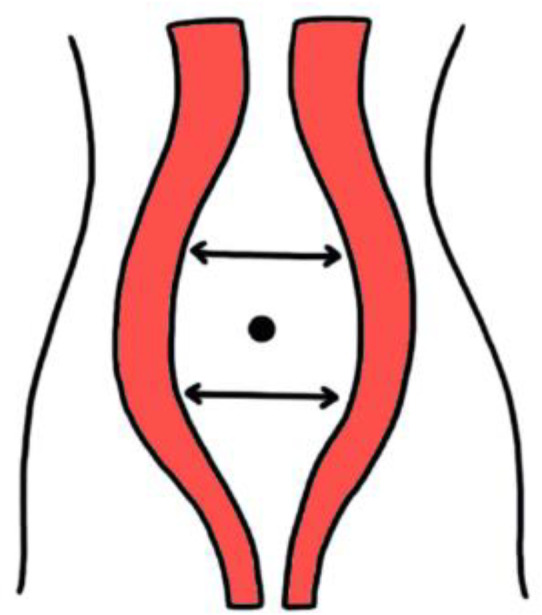
Measurement locations of inter-rectal space.

The probe was placed transversely along the midline of the abdomen, just above and below the navel (Figure 3). The decision to also perform subumbilical measurement was made to assess, for the first time in the literature, whether there is a difference between symptomatic and asymptomatic women in this area. The study did not require Ethical/Institutional review board approval because normal clinical practice has been followed [24]. Nevertheless, the Declaration of Helsinki was followed, and every patient signed a written informed consent. 

We used SPSS for Windows, version 17 (SPSS, Chicago, IL, USA) and GraphPad version 6 (GraphPad verison 6 Software, San Diego, CA, USA). Continuous variables were compared with Mann–Whitney or Student’s *t*-test as appropriate. Proportions of categorical variables were analyzed for statistical significance using Fisher’s exact test. Statistical significance was set at *p* < 0.05. 

## 3. Results

**Figure 4:**
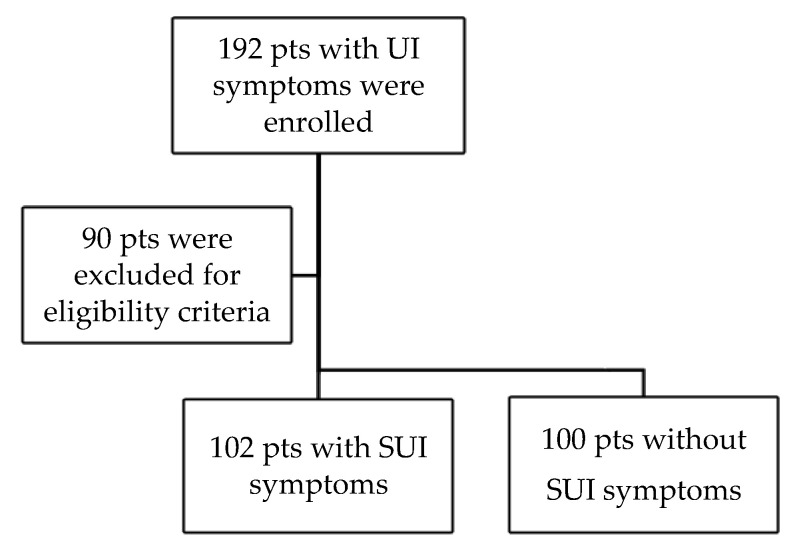
Flow chart of the total sample of patients across the study period.

During the study period, a total of 192 women with UI symptoms were enrolled. After applying the inclusion and exclusion criteria, 102 women remained eligible for the study group. One hundred women who did not report any symptoms of UI fit the inclusion criteria for group 2 (Figure 4).

The patient’s demographic characteristics, such as personal, medical, and gynecological history, were reported (Table 1). Median age was similar in the two groups (59.2 vs. 58.4; *p* = 0.87), and more than half of women were postmenopausal, 63.2% in SUI patients, and 66% in the control group (*p* = 0.72). We also found no significant differences in obstetric factors, such as parity (2% vs. 2%; *p* = 0.33), vacuum/forceps extraction (10% vs. 10%; *p* = 1), cesarean section (15% vs. 16%; *p* = 0.84) and macrosomy > 4000 gr (17% vs. 20%; *p* = 0.58) in each groups. 

In comparison, more hysterectomies [26 (25.5%) vs. 13 (13%)] were performed in group 1. Other from that, the two groups did not differ significantly in their characteristics. 

Table 2 shows the patient-reported outcomes and characteristics of UDS in SUI patients. The VAS scores obtained by the SUI group demonstrated a high degree of discomfort [VAS 7.5 (5–9)], as did the scores of the other questionnaires, 13 (9–16), 57 (46–67) and 27.5 (11–55) in the ICI-Q SF, UDI-6 SF and IIQ-7 SF, respectively. 

The inter-rectal distance above the umbilicus showed no significant difference between the two groups (2.12 ± 0.98 vs. 2.1 ± 0.77; *p* = 0.94) (Table 3). In contrast, the data from the sub-umbilical measurements showed a significant difference. Surprisingly, the asymptomatic group showed significantly greater (0.98 ± 0.9 vs. 1.33 ± 0.87 *p*-value = 0.009) inter-rectal distance compared to the symptomatic group.

## 4. Discussion

The aim of our case–control study was to find out whether DRA can affect women’s continence and clarify whether DRA-specific abdominal rehabilitation is needed in women with UI after childbirth. In this study, we have demonstrated that DRA is not a risk factor for the development of SUI; therefore, specific abdominal exercise for DRA should not be considered in the postpartum period.

The incidence of DRA in adult women is reported at about 28.4%. After age stratification, pregnancy and diabetes were found to be risk factors for DRA in young women, and obesity and diabetes were risk factors for DRA in older women [25]. DRA after childbirth is an issue that has been well demonstrated and examined in several studies. One in three women has an existing DRA one year after delivery [26]. The prevalence of DRA varies widely among studies because of the different definitions and cut-off values used for diagnosis. In addition, different measurement modalities such as finger or ultrasound assessment and different levels above or below the umbilicus to detect DRA, have been reported in the literature. Historically, DRA has been reported in 30–70% of pregnant women, and several studies have shown that it can persist into the early postpartum period in approximately 35–60% of women [27]. Other, more recent studies have reported a prevalence of DRA of 60%, 45%, and 33% at 6 weeks postpartum, 6 months, and 12 months postpartum, respectively [28]. However, a few studies in the literature have analyzed the impact of increased DRA on postpartum health disorders, such as low back pain, PFDs, and reduced abdominal muscle strength. A study by Gustavsson and Eriksson-Crommert [29], which aimed to describe how physiotherapists and midwives in primary healthcare perceive the phenomenon of increased inter recti distance (IRD) and its management in women after childbirth, concluded that there is no consensus among the health professionals on how to best approach increased IRD in the clinical setting. Their findings highlight the importance of further research to increase the professional knowledge base among healthcare providers and the urgent need for clinical guidelines in the management of DRA. 

An emerging postpartum rehabilitation (PPR) program, which explored the benefits of specific exercises including DRA rehabilitation on 403 participants in the first six weeks after delivery, showed a strong association between this program and reduction of postpartum depression symptoms and diastasis recti [30]. Based on this study, a comprehensive pelvic floor and DRA rehabilitation program in the early postpartum seems crucial to promote women’s physical and mental well-being. 

Liang et al. [31] in a randomised controlled trial with blinded assessment, evaluates the effect of a comprehensive rehabilitation program on closure of the rectus diastasis and quality of life in women after delivery. Sixty-six women were enrolled and assigned to the study group or control group by computerized randomization. Patients in the study group received electromyographic-biofeedback-assisted pelvic floor muscle training (BAPFMT) in combination with neuromuscular electrical stimulation (NMES) of the rectus abdominis, and patients in the control group underwent NMES of the rectus abdominis alone. The primary outcomes were changed in IRD and Short-Form Health Survey-36 (SF-36) scores, 6 weeks after the intervention. The authors found a significant decrease in IRD in the study group at 6 weeks [1.6 cm vs. control group 2.0; mean difference −0.4, 95% CI, −0.59 to −0.26] and a significant improvement in the study group compared with the control group [45.5 vs. 41.2; mean difference 4.3, 95% CI, 3.72–4.50] in the physical component summary of SF-36. They concluded that a postpartum program including BAPFMT for patients with DRA is feasible and improves the physical quality of life.

Thompson et al., in a small series of symptomatic women, showed altered muscle synergy between the pelvic floor and the abdominal wall. In fact, the symptomatic group was not able to perform a localized contraction of the pelvic floor muscle (PFM), but activated all the muscles of the abdominal wall, unlike the asymptomatic group [8]. This muscle replacement strategy reinforced the need to pay close attention to specificity when prescribing exercise programs for PFM, especially in the postpartum period. These results are also supported by Coldron et al. that examined ultrasound-guided DRA in women after childbirth [32]. In this cross-sectional and partial longitudinal study, the authors used ultrasound scanning to measure the changes in rectus abdominis muscles, such as cross-sectional area, thickness, width, and inter-recti distance, for the first year postpartum. They enrolled 115 postnatal women matched with 69 nulliparous women controls. They found that in all postnatal groups, recti abdominis was significantly thinner, wider and the inter-recti distance was significantly larger than in controls. Although, over 2 months postpartum, inter-recti distance decreased, it did not return to control values by 1 year. Liu et al. [33] investigated the association between SUI and DRA in patients with PFD at 6–8 weeks postpartum by vaginal palpation or pelvic floor biofeedback machine testing. They enrolled a total of 301 patients with PFD. Twenty-nine percent of patients had SUI and 31.9% (96 of 301) were diagnosed with DRA. They showed that a history of delivery (*p* = 0.012; odds ratio [OR] 1.982), vaginal childbirth with perianal lacerations or episiotomy (*p* = 0.016; OR, 2.187) were risk factors for SUI, besides high birth weight (weight > 4.0 kg, *p* < 0.001; OR, 14.507) being identified as a risk factor for DRA. In addition, a randomised control study of Kucukkaya et al. [34] showed the benefits of combining abdominal muscle training and PFMT in women with SUI. The authors enrolled 64 women with SUI allocated into the PFMT or pelvic floor muscle plus abdominal training (PFMT + AT) groups. They demonstrated that women who practiced both abdominal muscle training and PFMT achieved better results and faster recovery than women who practiced only pelvic floor training. In fact, the increase in the pelvic floor muscle activity was significantly greater for the PFMT + AT group than for the PFMT group (*p* < 0.05) and the negativity rate of the stress test at the 4th week was significantly higher for the PFMT + AT group (93.7%) than for the PFMT group (53.1%) (*p* < 0.001). 

Conversely, various studies have denied a relationship between SUI and DRA. 

In addition, there is no consensus among health professionals on the best way to treat DRA [35]. Two traditional treatments have been proposed to restore DRA: engagement of the transverse muscles of the abdomen or the rectus abdominis muscles. 

According to one of them, exercises of the transverse muscles of the abdomen (drawing-in exercise) are recommended, while all exercises that engage the rectus abdominis muscles are prohibited, as they have the potential to increase IRD (abdominal sit-up, curl-up, crunch exercise). Recently, results from various experimental studies have shown that curl-up leads to an immediate decrease in IRD, while in-drawing leads to an increase in IRD [35]. A cross-sectional study on 38 parous women has investigated the immediate effect of abdominal and PFM exercises on IRD in women with DRA using 2-dimensional real-time ultrasonography during rest and during eight randomly ordered different exercises. They found that head lift and twisted curl-up exercises decreased the IRD both above and below the umbilicus, whereas maximal in-drawing and PFM contraction exercises only increased the IRD below the umbilicus. However, they highlighted the need for high-quality randomized controlled trials to investigate if there is a long-term reduction in IRD by doing these exercises over time [36]. 

Fei et al. [21] demonstrated this in a retrospective cohort study which collected data from 229 postpartum women. Prevalence of DRA was 82.6% during the first year after delivery, and caesarean section [OR 3.48 (95% CI 1.42–8.56)] and multiple births [OR 3.20 (95% CI 1.59–6.45)] were identified as risk factors for DRA. The authors found no correlation between DRA and urinary incontinence or POP. Wang et al. in a cross-sectional study, compared pelvic floor muscle strength and the prevalence of UI and POP in 340 women with and without DRA at 6–8 weeks postpartum [15]. They showed that patients with DRA were not more likely to have weakened PFMS and increased UI or POP. 

A systematic review by Fuentes et al. [17], which included fourteen observational studies, showed an association between DRA and physical health and functioning, abdominal discomfort and body image, but no association with urinary incontinence. In addition, a randomized controlled trial by Gluppe et al. [37] assessed the effect of a postpartum training program on the prevalence of DRA. One hundred seventy-five primiparous women were randomly assigned to an exercise or control group. The authors showed no effect on DRA in women at 6 months postpartum (RR: 0.99 [0.71–1.38]) or at 12 months postpartum (RR: 1.04 [0.73–1.49]), when attempting abdominal training in addition to pelvic floor training. In our previous small series, we compared 35 primiparous women who had pure symptoms of SUI 6 months after delivery with 38 primiparous women without SUI symptoms. 

All measurements were taken by ultrasound examination with a probe placed transversely along the midline of the abdomen, just above the umbilicus. We found no statistically significant differences in the mean DRA value, [1.76 cm (±0.81 SD) in SUI group vs. 1.69 in control group (±0.79 SD)] [38]. Even though we excluded women over 45, the female population with the highest incidence of SUI symptoms, we concluded that an intervention on the abdominal muscles during pelvic floor rehabilitation treatment for SUI does not seem to be justified.

Another systematic review and meta-analysis on the effectiveness of abdominal and pelvic floor muscle exercise programs in the treatment of postpartum DRA was recently published [16]. The authors included seven randomized, controlled trials of 381 women who used transverse abdominal training and curl-up exercises as part of their intervention. The results provided very low-quality scientific evidence to recommend specific exercise programs in the treatment of DRA postpartum. 

Additionally, in this study, we did not find any association between DRA above the umbilicus and SUI. Surprisingly, a significant difference was found below the umbilicus. The data showed a greater inter-rectal distance in the asymptomatic group, not supporting our hypothesis that abdominal wall weakness such as in DRA could be considered a risk factor for SUI. The possible explanation could be related to the fact that women without SUI have a stronger pelvic musculature than patients with SUI, which results in abdominal pressure force lines perpendicular to the pelvis, leading to a greater widening of the DRA below the belly button. The strength of the study is the consistent methodology adopted in determining and measuring the DRA: we used ultrasound imaging, and the women were examined by only two physicians with great experience in PFD and in measuring DRA, which certainly led to a reduction of possible bias. In addition, we measured two different sites of the DRA to search for further differences with the same technique. Another point of strength was the number and the homogeneity of the sample size. Although data was collected at one site only, a substantial number of women were examined and included in the study. The limitation of the study is the lack of cut-off values for DRA, which generally also complicates the comparability of different studies.

## 5. Conclusions

The study shows that DRA is not a risk factor for SUI in women after childbirth. Surprisingly, the asymptomatic group showed significantly greater inter-rectal distance compared to the symptomatic group. Therefore, this study seems to demonstrate that no specific abdominal wall rehabilitation after childbirth is indicated.

## Figures and Tables

**Table 1:** Characteristics of patients.

	SUI Group	Control Group	*p* Value
	N = 102	N = 100	
Age, yr, median, (IQR)	59.2 (57.5–69)	58.4 (57.5–66.6)	0.87
BMI, kg/m^2^, median, (IQR)	24 (25–29)	23.4 (23–25.8)	0.72
Menopause, (%)	65 (63.7)	66 (66)	1
Parity, median, (IQR)	2 (1–4)	2 (1–4)	0.33
Vacuum/forceps extraction, (%)	10 (9.8)	10 (10)	1
Cesarean section, (%)	15 (14.7)	16 (16)	0.84
Macrosome > 4000 gr, (%)	17 (16.7)	20 (20)	0.58
Smoking habits, (%)	18 (17.6)	22 (22)	0.48
Previous Hysterectomy, (%)	26 (25.5)	13 (13)	0.03
Hormone Replace Therapy, (%)	7 (6.8)	8 (8)	0.79

**Table 2:** Patients reported outcomes and UDS characteristics in SUI patients.

Questionnaire	SUI Group
	(m/IQR)
ICI-Q SF	13 (9–16)
UDI-6 SF	57 (46–67)
IIQ-7 SF	27.5 (11–55)
VAS	7.5 (5–9)
MUCP (cmH_2_O)	47 (31–64)
I-OpenP (cmH_2_O)	8 (4–15)
VLPP (cmH_2_O)	60 (21–82)

MUCP = Maximum Urethral Closure Pressure; I-OpenP = Intravesical Opening Pressure; VLPP = Valsalva Leak Point Pressure.

**Table 3:** Diastasis Recti Abdominis measurements in group 1 and 2.

	SUI Group	Control Group	*p* Value
	N = 102	N = 100	
DRA superior	2.12 ± 0.98	2.1 ± 0.77	0.94
DRA inferior	0.98 ± 0.9	1.33 ± 0.87	0.009

## Data Availability

Data can be requested from the authors.
